# Long-Lasting Immune Protection and Other Epidemiological Findings after Chikungunya Emergence in a Cambodian Rural Community, April 2012

**DOI:** 10.1371/journal.pntd.0004281

**Published:** 2016-01-11

**Authors:** Beatriz Galatas, Sowath Ly, Veasna Duong, Kathy Baisley, Kunthy Nguon, Siam Chan, Rekol Huy, Sovann Ly, Sopheak Sorn, Leakhann Som, Philippe Buchy, Arnaud Tarantola

**Affiliations:** 1 Institut Pasteur du Cambodge, Phnom Penh, Cambodia; 2 London School of Hygiene and Tropical Medicine, London, United Kingdom; 3 Ministry of Health, Phnom Penh, Cambodia; Pediatric Dengue Vaccine Initiative, UNITED STATES

## Abstract

The East/Central/South African genotype of Chikungunya virus with the E1-A226V mutation emerged in 2011 in Cambodia and spread in 2012. An outbreak of 190 cases was documented in Trapeang Roka, a rural village. We surveyed 425 village residents within 3–4 weeks after the outbreak, and determined the sensitivity and specificity of case definitions and factors associated with infection by CHIKV. Self-reported clinical presentation consisted mostly of fever, rash and arthralgia. The presence of all three clinical signs or symptoms was identified as the most sensitive (67%) and specific (84%) self-reported diagnostic clinical indicator compared to biological confirmation by MAC-ELISA or RT-PCR used as a reference. Having an indoor occupation was associated with lower odds of infection compared with people who remained at home (adjOR 0.32, 95%CI 0.12–0.82). In contrast with findings from outbreaks in other settings, persons aged above 40 years were less at risk of CHIKV infection, likely reflecting immune protection acquired when Chikungunya circulated in Cambodia before the Khmer Rouge regime in 1975. In view of the very particular history of Cambodia, our epidemiological data from Trapeang Roka are the first to support the persistence of CHIKV antibodies over a period of 40 years.

## Author Summary

After first being identified in the 1950s and spreading from Africa in the 1960s, a new pandemic strain of Chikungunya virus emerged in East Africa and the Indian Ocean in 2004–2005, progressing to Asia. Cases have since been described in previously unaffected territories, as well as regions where Chikungunya transmission was never interrupted. Chikungunya circulated in Cambodia in the 1960s and 1970s until the tragic historical events that followed the civil war. After nearly 40 years of absence of the virus, the new pandemic strain was first detected in 2011. We undertook a field investigation of a Chikungunya outbreak in Cambodia in 2012. The usefulness of clinical signs for diagnosis of infection is discussed. Unlike studies from other settings, we show that older age was associated with a lower risk of Chikungunya infection, even after behavioural and occupational factors have been taken into account. This is the first evidence suggesting that infection in the 1960s and 1970s likely provided long-lasting cross-protection against this new strain. These findings, which will be further explored in the laboratory, are important to understand immunity against Chikungunya and to predict future epidemics and public health needs.

## Introduction

Chikugunya is caused by an alphavirus transmitted by the bite of *Aedes* mosquitoes. In humans, it is mostly a self-limiting illness marked with debilitating joint pains but severe illness occurs in about 1 clinical case in 1000 [1]. Although it may have circulated since the late 1800s [2], the chikungunya virus (CHIKV) was first detected in Africa in 1952 [3]. The Asian strain spread through Asia in the 1960s causing a series of outbreaks throughout the region, including Cambodia. After several decades of absence, CHIKV re-emerged in the early 2000s [3–5], with large outbreaks of significant public health concern in Asia and Africa. In 2005, a major epidemic in La Réunion island [6] displayed different epidemiological characteristics than previous outbreaks, with a higher attack rate and causing a number of deaths. Genetic analysis attributed this outbreak to a mutated strain of the East/Central/South African (ECSA) strain of CHIKV bearing the E1-A226V and other mutations on the E2 glycoprotein gene [7,8], termed the Indian Ocean Lineage (IOL) strain [8]. Subsequently, outbreaks of the IOL strain have been recorded in the Indian Ocean [9–11], South- [12], Southeast- [13–17] and East Asia [18] and the Pacific [19]. The first outbreak in a temperate country was recorded in 2007 [20], and cases have been detected in Arabia [21,22]. In 2013, another CHIKV strain, this time of Asian lineage [23] stormed through the Americas, causing over 1.5 million suspected or confirmed cases to date [24–27]. That outbreak is still ongoing. Chikungunya poses a real and imminent threat to all yet unaffected areas where *Aedes aegypti* or *Aedes albopictus* are present, including various regions of Europe [28], the USA [29], Brazil [30] or Australia [31].

Chikungunya re-emerged in Cambodia in 2011 [16], when the IOL strain of CHIKV was identified, never previously recorded in Cambodia. In this article, we explore the epidemiological and clinical characteristics of a 2012 outbreak in Trapeang Roka village of Kampong Speu province [17], Cambodia, including an analysis of the sensitivity and specificity of clinical signs and their combination, as well as the association of various factors with the risk of CHIKV infection.

## Methods

### Field investigation

The method, dates and duration of the outbreak investigation in Cambodia have been detailed elsewhere [17]. Briefly, a one-day outbreak investigation was conducted in 2012, three to four weeks after the onset of the CHIKV outbreak in Trapeang Roka village caused by an ECSA strain with the E1-A226V mutation, using a standardized, anonymized questionnaire. All village residents were eligible for the investigation; consenting individuals were interviewed regarding socio-demographic characteristics and information on potential risk factors for exposure and were screened for CHIKV. Participants were asked about self-reported clinical features (fever, arthralgia and skin rash) using a short questionnaire and the Wong-Baker Pain Rating scale. For children who were too young to answer, parents/guardians were asked to answer for the child. Interviews were conducted by teams from the Epidemiology and Public Health unit of the Institut Pasteur du Cambodge (IPC) and other teams.

### Laboratory methods

Dried blood spots were obtained from consenting participants to test for CHIKV IgM [32], as well as recent infection by dengue (DENV) and Japanese encephalitis B (JEV) viruses using antibody capture enzyme-linked immunosorbent assay (MAC-ELISA) [33,34]. Participants with ongoing fever were also tested by quantitative real-time reverse transcriptase PCR (qRT-PCR) for CHIKV as well as DENV and JEV. Participants (N = 91) with current or very recent signs or symptoms were also screened for malaria by PCR (all negative). The laboratory methods used were detailed in the previous report [11].

### Statistical / epidemiological analysis

#### Sensitivity / specificity

Clinical signs or symptoms were tabulated individually and in combination (the presence/absence of a combination of signs or symptoms) against laboratory-confirmed infection with CHIKV through MAC-ELISA or qRT-PCR. The sensitivity, specificity, positive predictive value (PPV), and negative predictive value (NPV) of individual and combined signs or symptoms were calculated, to assess their value as diagnostic tools for CHIKV.

#### Statistical analysis

Factors associated with CHIKV infection were assessed using random effects logistic regression to estimate odds ratios (ORs) and 95% confidence intervals (CI), adjusted for correlation/clustering within households. Statistical significance was evaluated using likelihood-ratio tests (LRTs).

A multivariate model was built adjusting for age as an *a priori* risk factor. All variables that associated with CHIKV infection at p<0.15 in the age-adjusted analysis were included in the multivariate model.

Given the re-emergence of CHIKV in Cambodia, it was considered relevant to explore the shape of the relationship of participant age with CHIKV infection. The association of age as a continuous variable with the log odds of CHIKV infection was assessed using fractional polynomials, using a set of defined powers (–2, –1, –0.5, 0.5, 1, 2 and ln(x)) and a maximum of two power terms in the model. Age was first scaled by dividing by 10 before fitting the models. The differences in model deviances were compared to select the best model. Predicted log ORs from each model were transformed into risks through the equation [exp(OR)/(1+exp(OR)], and predicted probability plots were created from the univariate and multivariate models to observe associations of age to CHIKV infection. Probability plots from the multivariate model fixed the other model covariates at their mean values. All analyses were performed using the STATA 11 statistical package (StataCorp, College station, TX, USA).

### Ethics statement

The investigation was undertaken as part of an urgent public health investigation of emerging CHIKV in Cambodia, with a National Ethics Committee waiver under the authority of the Ministry of Health. After explaining the investigation in Khmer, participants were asked for written informed consent to undergo an interview and a fingerprick for blood sampling. Participants received advice on how to reduce their risk of CHIKV infection and received their laboratory results at a later visit. All data collected were anonymised, with no personal identifiers.

## Results

In March 2012, Trapeang Roka consisted of 134 households with 695 inhabitants, mainly rice field workers and factory workers. All persons who were available on the day of the outbreak investigation were approached to be interviewed; there were no refusals. In total, 425 participants (61.1% of the village) from 98 households (73.1%) were surveyed. The global attack rate was 44.7% (190/425) based on IgM (n = 188) or PCR (n = 2) testing.

### Sensitivity and specificity of clinical signs

Findings are presented in Table 1. The combined signs of fever, arthralgia and skin rash was 67% sensitive as a CHIKV diagnostic tool compared to the reference laboratory diagnostic test. The combination of all three signs or symptoms also identified the highest proportion of laboratory confirmed CHIKV infections during the outbreak (PPV = 77.3%), while those who did not experience these combination of signs or symptoms represented 76.3% (NPV) of the truly uninfected. The specificity of individual sign or symptoms, or combination thereof, was higher than the sensitivity, with between 84.4% to 98.7% of those without the sign or symptom being correctly identified as CHIKV IgM-negative, depending on which combination of signs or symptoms was considered.

**Table 1:** Self-reported clinical signs of CHIKV E1-A226V infection, and their diagnostic sensitivity/specificity and predictive values, Trapeang Roka, 2012.

Self-reported clinical signs		CHIKV IgM+ (%)*	CHKIV IgM—(%)	Total	p-value from χ^2^ Test	Se*	Sp*	PPV*	NPV*
Fever only	Present	12 (6)	26 (11)	38 (9)	0.09	6.4%	89.0%	31.6%	54.5%
	Absent	176 (94)	211 (89)	387 (91)					
Arthralgia only	Present	8 (4)	25 (11)	33 (8)	0.01	4.3%	89.5%	24.2%	54.1%
	Absent	180 (96)	212 (89)	392 (92)					
Rash only	Present	3 (2)	3 (1)	6 (1)	0.79	1.6%	98.7%	50.0%	55.8%
	Absent	185 (98)	234 (99)	419 (99)					
Fever and Rash	Present	4 (2)	5 (2)	9 (2)	0.99	2.1%	97.9%	44.4%	55.8%
	Absent	184 (98)	232 (98)	416 (98)					
Fever and Arthralgia	Present	19 (10)	31 (13)	50 (12)	0.35	10.1%	86.9%	38.0%	54.9%
	Absent	169 (90)	206 (87)	375 (88)					
Rash and Arthralgia	Present	6 (3)	7 (3)	13 (3)	0.88	3.2%	97.0%	46.2%	55.8%
	Absent	182 (97)	230 (97)	412 (97)					
ALL	Present	126 (67)	37 (16)	163 (38)	<0.001	67.0%	84.4%	77.3%	76.3%
	Absent	62 (33)	200 (84)	262 (62)					
Total		188	237	425					

*Se: sensitivity; Sp: Specificity; PPV: positive predictive value; NPV: negative predictive value.

### Association between age and risk of infection

**Fig 1:**
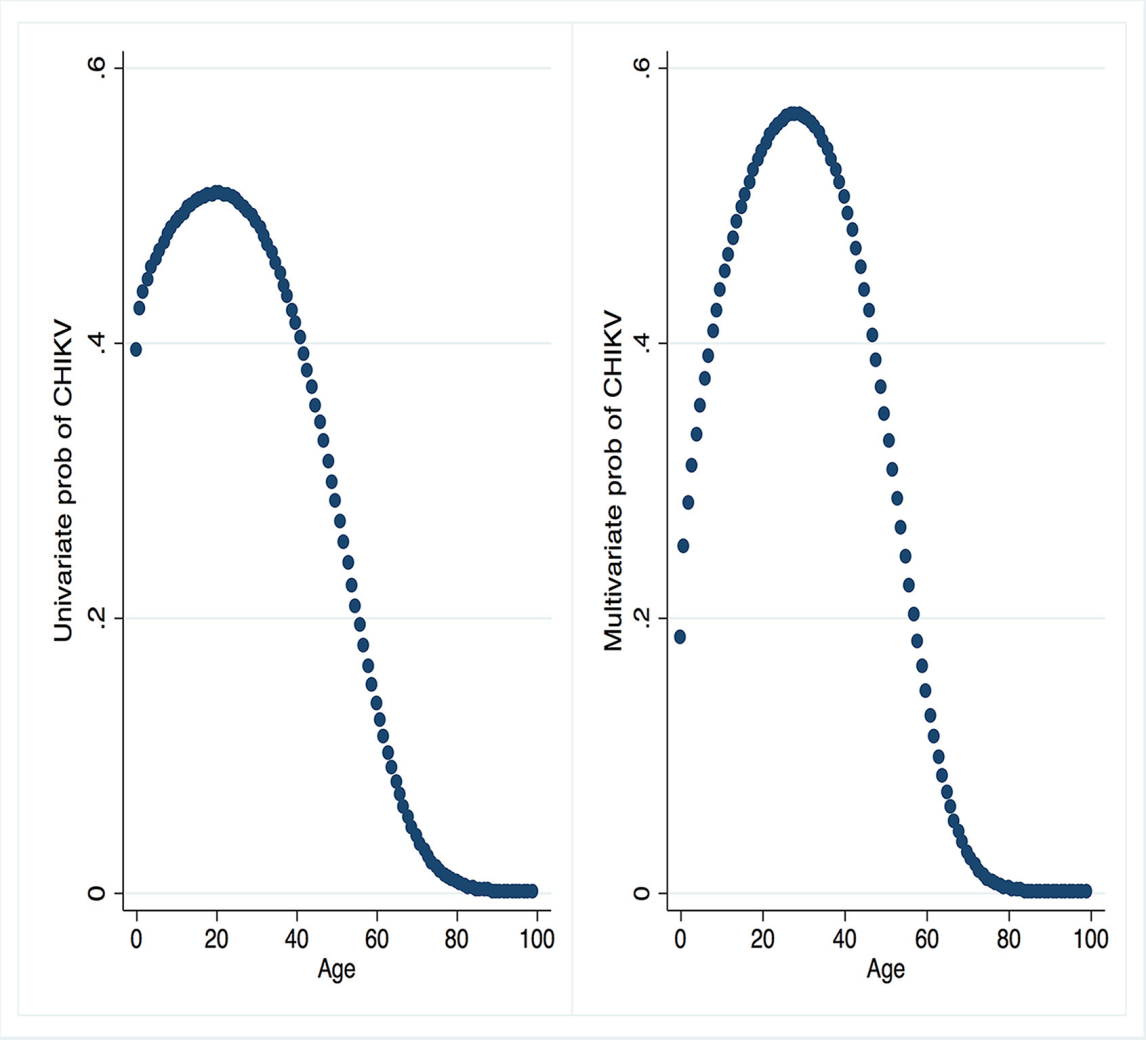
Association curve between age and risk of CHIKV infection in univariate analysis and after multivariate analysis, using random effects logistic regression to account for correlation within households, Trapeang Roka, Cambodia, 2012.

Age was strongly associated with laboratory-confirmed CHIKV infection in both the univariate and multivariate models (p<0.001); the shape of the association is shown in Fig 1. The predicted risk of CHIKV peaked at 50% among those age 15 to 26 years (born between 1986 and 1997) and then declined abruptly, reaching a low of 0.1% by age 87. After adjusting for gender and occupation, the predicted adjusted risk of CHIKV was highest (58%) among 24–32 year-olds (born between 1980 and 1988).

### Other risk factors associated with CHIKV infection

Table 2 shows the association of selected risk factors with laboratory-confirmed CHIKV infection, none of which showed strong evidence of an association, likely because of the relatively small sample size. There was some evidence that males were more likely to be infected than women (adjusted (adj) OR = 1.61; 95%CI 0.93–2.77; p = 0.09). There was also some evidence of an association with occupation, with CHIKV infection being less likely among participants who had an indoor occupation compared with those who stayed at home (adjOR 0.32, 95%CI 0.12–0.84, p = 0.07). There was also some evidence that those who had a healthcard–a government-issued certificate to allow the poorest Cambodians access to free healthcare–were at lower risk of infection. There was no evidence of an association of CHIKV infection with level of education.

**Table 2:** The association of selected risk factors to CHIKV infection (after adjusting for age, sex, occupation and healthcard), Trapeang Roka, Cambodia, 2012.

Risk Factors	Total	CHIKV Positive	Crude OR	LRT-p-value	Adjusted OR §	LRT p value
	(column %)	(row %)	(95% CI)		(95% CI)^§^	
Total (N = 425)	425	190 (45)				
Age						
<1	10 (2)	4 (40)		<0.001		<0.001
1–5	39 (9)	15 (38)				
6–20	153 (36)	83 (54)	*p*1:1.48 (0.72–3.03) ^1^		*p*1:3.48 (1.28–9.48) ^1^	
21–40	126 (30)	60 (48)	*p*2:0.99 (0.98–1.00) ^1^		*p*2:0.98 (0.98–0.99) ^1^	
41–60	67 (16)	25 (37)				
61+	30 (7)	3 (10)				
Sex						
Females	236 (56)	91 (39)	1	0.01	1	0.09
Males	189 (44)	99 (52)	1.95 (1.2–3.2)		1.61 (0.93–2.77)	
Education						
None	118 (28)	38 (32)	1	0.05	1	0.53
Primary	230 (54)	112 (49)	2.09 (1.1–3.9)		1.53 (0.67–3.48)	
Secondary or higher	77 (18)	40 (53)	2.03 (0.9–4.5)		1.17 (0.42–3.27)	
Occupation						
Stay at home	134 (32)	58 (43)	1	0.09	1	0.07
Student	89 (21)	52 (58)	1.82 (0.9–3.8)		1.00 (0.45–2.22)	
Outdoor activities	111 (26)	41 (37)	0.72 (0.4–1.4)		0.46 (0.18–1.19)	
Indoor activities	91 (21)	39 (43)	0.81 (0.4–1.7)		0.32 (0.12–0.84)	
Owning a Healthcard^2^						
No	408 (96)	189 (46)	1	0.02	1	0.04
Yes	15 (4)	1 (7)	0.04 (0.02–0.8)		0.05 (0.00–1.07)	

§ OR adjusted for age, sex and occupation and healthcard, from random effects logistic regression to account for correlation within household.

^1^Best fitting fractional polynomial model for age: p1 = (age/10)^0.5^; p2 = (age/10)^3 2^Healthcard: a nominative card issued by the authorities to the poorest segment of the population to benefit from free healthcare

## Discussion

Our findings show that in a country such as Cambodia that is highly endemic for dengue, the most sensitive and specific way of clinically diagnosing CHIKV infection based on patients’ self-reported signs is with the simultaneous presentation of fever, joint pain and rash. This clinical case definition of CHIKV can be used to detect outbreaks, especially in age groups not usually affected by dengue, and for surveillance of epidemic trends. It cannot, however, guide individual patient management of severe cases or help precisely measure caseload, and may be of limited use in a setting with seasonal dengue outbreaks. Access to laboratory confirmation is therefore essential for patient management, for intermittent human and virological surveillance, and to confirm the beginning and end of an outbreak.

Few data are available on the risk factors associated with infection by the ECSA CHIKV strain, and most previous studies have been conducted in endemic settings or during emergence of the virus. Previous studies have identified older age [6,35,36], gender–male [37] or female [3,6,38], low education [37], housework [3] and low socio-economic status [37] as main risk factors for CHIKV infection, although findings vary between studies and populations. Faced with the large-scale emergence of CHIKV, Cambodian health authorities and their partners invested precious time, effort and scarce resources to document the outbreak in a rural community setting [17] as well as investigate risk factors for CHIKV infection. In this study in Trapeang Roka, we found some evidence that males were more likely to be infected by CHIKV. Occupation also seemed to play a role: indoor activities outside the home (factory work, for example) seemed to provide protection against CHIKV infection compared to staying at home. This is likely to be related to a reduction in *Aedes aegypti* exposure outdoors compared with people who stay at home and may spend time indoors. A lack of association of occupation with CHIKV infection has been observed in Mayotte [37], while other studies have observed housework as a risk factor [3] or a protective factor for CHIKV [39]. This is likely due to differences in outbreak settings and environments that determine the presence or absence of CHIKV positive individuals and the distribution of *Aedes* mosquitoes.

An elegant study of limited scope conducted in Greece demonstrated that antibodies against dengue virus can last a lifetime [40]. The duration of immunity conferred by CHIKV infection is unknown. In our study, villagers younger than 40 were at higher risk of CHIKV infection after adjustment for other socio-demographic variables, while those over 40 had the lowest risk of infection. The opposite might have been expected, since older persons tend to remain around the home so would have higher risk of exposure to *Aedes* mosquitoes. Furthermore, the observed age distribution is inverse to what has been shown in all other major outbreaks caused by the ECSA CHIKV strain, such as those in La Réunion island [6], India [35] or Malaysia [36], where older age was identified as a strong risk factor for the infection.

The most likely explanation of the age pattern of infection in Trapeang Roka is the presence of pre-existing antibodies among the older population who were exposed to the Asian CHIKV lineage during the early 1960s [41–43]. Civil war erupted in Cambodia in 1968 and lasted until the April 1975 Khmer Rouge takeover and terror regime, during which people were forbidden to travel [44]. As one of many consequences, large-scale circulation of CHIKV in Cambodia was no longer detectable [16,17]. The Asian strain of CHIKV continued to circulate in Asia, especially Thailand [45]. Virological testing for dengue-like syndromes in Cambodia at the Institut Pasteur du Cambodge was initiated in 2000, and systematically and actively sought but never detected CHIKV until 2011, when it re-emerged [16]. Our data show that the risk of CHIKV infection was highest in people who were born since 1975. This provides compelling evidence that the previously circulating Asian CHIKV lineage very likely provided immune protection against the new ECSA CHIKV strain bearing the E1-A226V mutation and that this immunity has persisted for 40 years.

Our study has limitations. For the risk factor analysis, our sample size is small, so our power to detect significant associations with risk factors is not high. There may be some multicolinearity (redundancy) between the variables of age and occupation, and there may be some residual confounding because of imperfectly measured variables. Also, the decrease in risk of CHIKV infection among villagers aged above 40 could be due to behavioral factors that we did not measure which therefore could not be adjusted for in the analysis. However, the observed effect is strong, the risk falls rapidly after age 40 (not only in the elderly) and persons of all ages live in close proximity with very similar lifestyles and exposure to *Aedes* mosquitoes. The broad range of risk factors examined in the study would likely have provided a proxy for any other strong, undocumented exposure. Lastly, some village residents may have been missed as they were away at work, thereby overestimating the risk among 20–40 year olds. We are confident, however, that this does not seriously challenge the validity of our data: although the number of interviews was limited due to the one-day nature of the investigation, all persons approached agreed to participate and over 60% of residents, worker or not, and 70% of households, were interviewed and sampled.

For the analysis of the sensitivity and specificity of clinical signs or symptoms, we used self-declared signs or symptoms rather than those observed by medical staff. There is therefore a possibility of response bias. Several studies undertaken at the Epidemiology and Public Health Unit of the Institut Pasteur du Cambodge have shown how labile answers to non-objective questions (signs or symptoms) can be, to answer the presumed wishes of the interviewer. In order to minimize this, questionnaires were administered carefully by trained native Khmer-speaking interviewers. Finally, the sensitivity and specificity of the MAC-ELISA method used for measuring anti-CHIKV IgM are unknown.

### Conclusion

Studies of emergent pathogens in the laboratory or in the hospital setting are very important to identify risk factors for increased circulation (such as CHIKV mutations [7,8]) or severe clinical forms [1] of infection. However, the findings that will guide surveillance, risk assessment and public health prevention and response should originate mainly from careful epidemiological documentation in the community of the true health burden of disease, risk factors associated with infection and the performance of clinical case definitions. Our data show that current case definitions are both sensitive and specific enough to guide initial epidemiological assessments but must be complemented by virological tests performed by experienced laboratories. Importantly, these data from Cambodia—with its unique and dramatic history—strongly suggest that immunity against CHIKV may last several decades, an important element for future risk assessment in non-endemic settings and as this virus spreads across the New World and the Pacific. These epidemiological findings will be verified in the laboratory by testing immunity against the CHIKV strain which circulated in the Mekong Region in the 1960s.
